# *Salmonella* meningitis bacteremia in two‐week neonate: A rare and devastating disease

**DOI:** 10.1002/pdi3.2510

**Published:** 2025-03-12

**Authors:** James S. Bassett, Jerrin George, Michelle Cherian

**Affiliations:** ^1^ College of Medicine and Life Sciences University of Toledo Toledo Ohio USA

## 1 INTRODUCTION

Meningitis is an inflammation of the protective membranes surrounding the brain and spinal cord, typically arising from bacterial, viral, or fungal infections. While bacterial meningitis is a well‐documented and serious condition, the association with *Salmonella* infection presents a rare and distinctive challenge in its medical management. This unique manifestation of *Salmonella* meningitis accounts for a mere 0.8%–6.0% of all bacterial meningitis cases,^1^ underscoring the infrequency of this microbial etiology.

*Salmonella*, recognized primarily for causing gastrointestinal infections, reveals a propensity for atypical presentations such as meningitis. The overall mortality rate of *Salmonella* meningitis is a staggering 18%,^2^ further amplifying the life‐threatening consequences of this condition. Neurological complications arising from *Salmonella* meningitis compound the complexity of the disease. Empyemas, intracerebral abscesses, intracranial hemorrhages, and hydrocephalus constitute a formidable array of challenges faced by clinicians managing patients with this rare manifestation. Importantly, these complications are frequently associated with a poorer prognosis, exacerbating the urgency for a comprehensive understanding of the disease manifestation, early detection, and effective management with appropriate workup and imaging.

Our discussion involves a 2‐week‐old male who presented with fever and mild distress secondary to *Salmonella* meningitis bacteremia. The intricacies of this case emphasize the potential severity and rapid progression of *Salmonella* meningitis, serving as a reminder of the multifaceted nature of the challenges posed by this uncommon but potentially devastating manifestation, emphasizing the need to include *Salmonella* meningitis into one's differential diagnosis and obtain an urgent diagnostic workup. Furthermore, the limited evidence on the management of *Salmonella* meningitis merits further investigation on specific therapeutic guidelines to improve patient outcomes.

### 1.1 Case presentation

**FIGURE 1:**
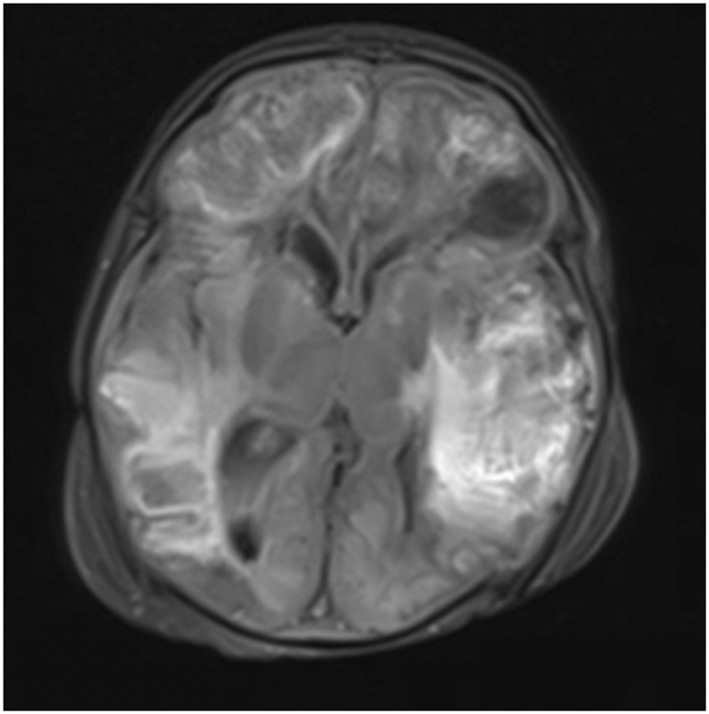
MRI brain with contrast flair axial view revealing significant progression of disease with extensive multifocal bilateral intraparenchymal hemorrhage.

A 10‐day old Caucasian male presented to the emergency department (ED) with a fever of 102°F. The patient was born by C‐section at full term (39 weeks, 6 days) with no maternal infection present and was found healthy at his one‐week appointment. Physical examination revealed the patient to be warm and inconsolable, but was otherwise unremarkable. The workup of the patient revealed a white blood count (WBC) of 4.7, but otherwise negative respiratory pathogen panel (RPP), complete blood count (CBC), and comprehensive metabolic panel (CMP). RPP ruled out a variety of strains of adenovirus, coronavirus, human metapneumovirus, rhinovirus/enterovirus, influenza, parainfluenza, respiratory syncytial virus, *Bordetella pertussis*, *Chlamydia pneumoniae*, *Mycoplasma pneumoniae*, and SARS COVID 2. The patient's mother was group B *Strep* (GBS) negative at the time of hospital admission. Lumbar puncture of the patient was attempted, but was unsuccessful due to patient's critical status. The patient was given one dose of ceftazidime, gentamicin, acyclovir, and two doses of ampicillin in the ED. Retrieved blood cultures grew gram negative rods of *Salmonella* in 3 out of 9 cultures acquired during the hospital course which was limited to the evaluation of aerobic bacteria. The course was also complicated by multiple strains of Multidrug‐resistant *Staph* that were found 5 days into the hospital stay. Computed tomography (CT) of brain without contrast revealed abnormal attenuation throughout the right frontal and parietal lobes with sulcal effacement, loss of gray‐white differentiation, and small left frontal axial fluid‐filled collection (Figure S1). The patient was then transferred to the pediatric intensive care unit (PICU). Repeat CT brain without contrast 2 days later revealed significant progression of disease with extensive multifocal bilateral intraparenchymal hemorrhage throughout the supratentorial brain (Figure 1). There were also surrounding regions of hypoattenuation noted reflecting superimposed areas of vasogenic edema (Figure S2). Ventricular catheter was placed using right frontal convexity approach (Figure S3). Patient prognosis was very poor upon admission. His course peaked in severity 3 days into treatment as the child sustained multiple seizures, had a sodium of 161 mmol/L, WBC count of 25.2, and platelet count of 16. Despite managing seizures with levetiracetam and stabilizing electrolytes through fluid replacement, several subsequent spontaneous breathing trials were unsuccessful and the patient expired 3 weeks following initial hospital admission.

## 2 DISCUSSION

Although *Salmonella* does not commonly cause meningitis, incidence rates have progressively risen since the mid‐2000s making it a growing public health concern. National surveillance data suggests that *Salmonella* meningitis necessitates hospitalization, but hospitalization alone may not be sufficient for fortuitous outcomes. In fact, there are significant issues related to the infection including high mortality, high treatment failure rates, and rising morbidity rates in survivors. Rates of amino acid uptake to the brain are highest in the first week, suggesting that early infants are especially susceptible to an accelerated meningeal spread due to increased permeability of the blood–brain barrier.^3^ Other factors that facilitate spread include birth trauma, underdeveloped cellular immunity, or transmission from an affected mother.^3^ Common complications in small samples of infantile *Salmonella* meningitis include hydrocephalus and subdural effusion, in which each have a cure rate of roughly 50% even with surgical intervention.^2^ Although limited in evidence, current treatment guidelines for *Salmonella* meningitis in infants include an antibiotic regimen containing a third‐generation cephalosporin and a fluoroquinolone. However, studies have shown that the *Salmonella* pathogen is developing increase resistance against fluoroquinolones and require alternative treatments with trimethoprim‐sulfamethoxazole or amicacin,^4^ as opposed to the antibiotic regimen given to our patient. Due to the rapid progression of symptoms and uncommon presentation in this case report, there is merit for further investigation of management and early detection of *Salmonella* meningitis to improve patient outcomes. The poor prognosis and rapid deterioration of this disease necessitates physicians to maintain a high clinical suspicion when initiating diagnostic assessments toward the workup of pediatric *Salmonella* meningitis in efforts to improve patient prognosis.

## 3 CONCLUSION

This case report underscores the critical nature of *Salmonella* meningitis in infants, a rare yet increasingly recognized manifestation of *Salmonella* infection. Early diagnosis and prompt initiation of treatment for *Salmonella* meningitis are essential to prevent complications, reduce mortality rates, control contagion, and improve overall patient outcomes. Timely intervention is crucial in mitigating the potentially severe consequences of this bacterial infection on the central nervous system. The limited evidence and evolving resistance patterns of *Salmonella* against certain antibiotics underscore the necessity for further research to refine and enhance therapeutic guidelines. Further investigation is required to improve patient outcomes for patients who contract *Salmonella* meningitis.

## AUTHOR CONTRIBUTIONS

**James S. Bassett**: Conceptualization; project administration; interpretation of data; writing—original draft. **Jerrin George**: Conceptualization; project administration; investigation; writing—original draft. **Michelle Cherian**: Writing—review and editing; interpretation of data.

## CONFLICT OF INTEREST STATEMENT

The authors declare no conflicts of interest.

## ETHICS STATEMENT

Hereby, I James Bassett, Jerrin George, and Michelle Cherian consciously assure that for *Salmonella* Meningitis Bacteremia in Two‐Week Neonate: A Rare and Devastating Disease, the following is fulfilled: This material is the authors' own original work, which has not been previously published elsewhere. The paper is not currently being considered for publication elsewhere. The paper reflects the authors' own research and analysis in a truthful and complete manner. The paper properly credits the meaningful contributions of coauthors and coresearchers. The results are appropriately placed in the context of prior and existing research. All sources used are properly disclosed (correct citation). All authors have been personally and actively involved in substantial work leading to the paper and will take public responsibility for its content. Hospital review board exempts this case report from ethics committee approval.

## Supporting information

Supporting Information S1

## Data Availability

The data that support the findings of this study are available on request from the corresponding author.
