# Supplementary material for: Salmonella meningitis bacteremia in two‐week neonate: A rare and devastating disease
Source: Pediatr Discov. 2025 Mar 12;3(1):e2510. doi: 10.1002/pdi3.2510 (PMC12118101; doi:10.1002/pdi3.2510)
Supplement: Supplementary file 1 — Supporting Information S1 [file PDI3-3-e2510-s001.docx]

**Salmonella Meningitis Bacteremia in Two-Week Neonate: A Rare and Devastating Disease- Supplemental Information**

Supplemental Figures:


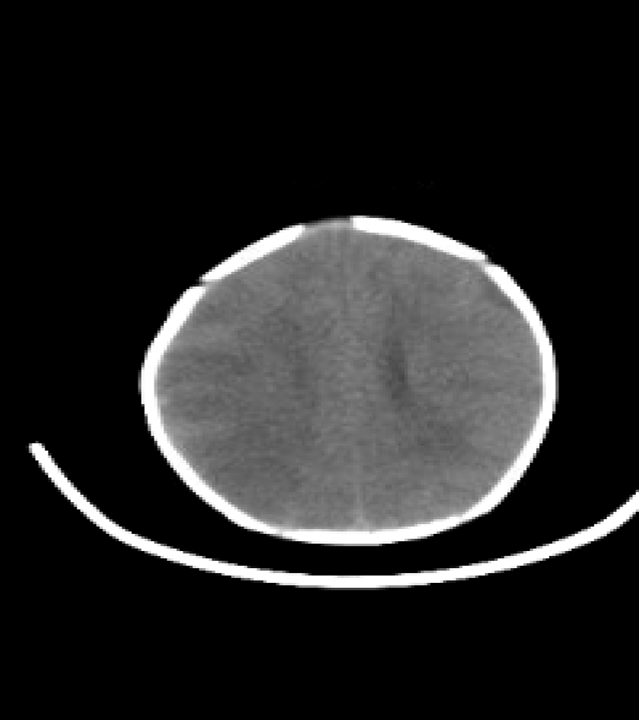


**Supplemental Figure 1: Initial CT Brain w/o Contrast revealed abnormal attenuation throughout the right frontal and parietal lobes. Loss of gray-white differentiation.**


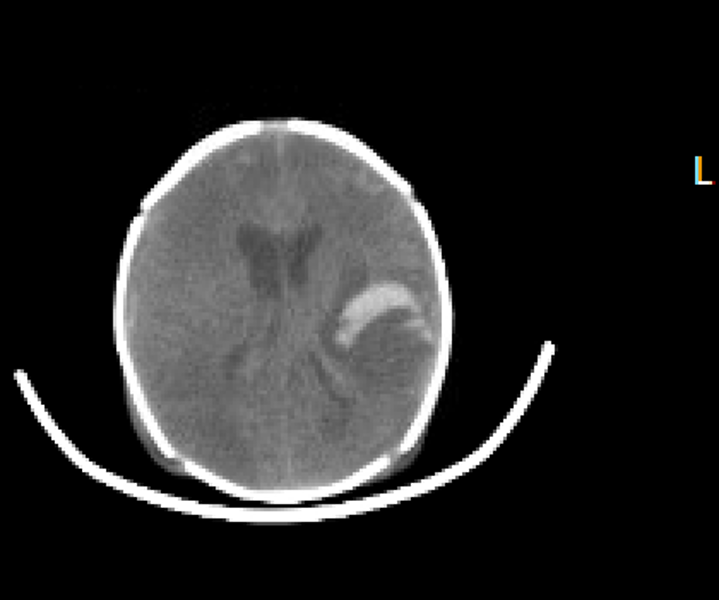


**Supplemental Figure 2: Repeat CT brain w/o contrast Axial view- surrounding regions of hypoattenuation reflecting superimposed areas of vasogenic edema.**


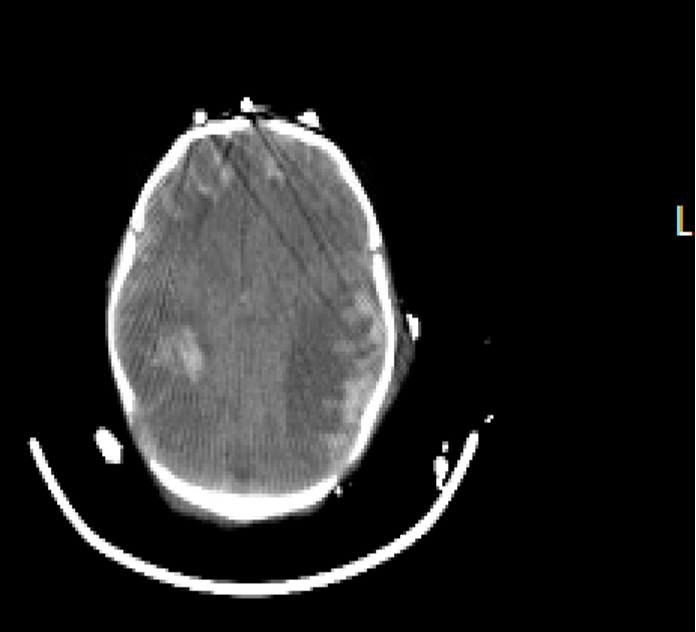


**Supplemental Figure 3: Repeat CT brain w/o contrast axial view revealing ventricular catheter placed using right frontal convexity approach.**
